# “Come on! He Has Never Cooked in His Life!” New Alternative Masculinities Putting Everything in Its Place

**DOI:** 10.3389/fpsyg.2021.674675

**Published:** 2021-05-05

**Authors:** Rosa Valls-Carol, Antonio Madrid-Pérez, Barbara Merrill, Guillermo Legorburo-Torres

**Affiliations:** ^1^Department of Theory and History of Education, University of Barcelona, Barcelona, Spain; ^2^Department of Political Science, Constitutional Law, and Philosophy of Law, University of Barcelona, Barcelona, Spain; ^3^Centre for Lifelong Learning, University of Warwick, Coventry, United Kingdom; ^4^Department of Pedagogy, University of Rovira i Virgili, Tarragona, Spain

**Keywords:** communicative acts, masculinities, discourses, domestic work, reprimands, gender

## Abstract

Communicative acts of some women are perpetuating the dominance that DTM (Dominant Traditional Masculinities) have over both women and OTM (Oppressed Traditional Masculinities). Some women use language in a disdainful manner to reprimand oppressed men’s behavior in daily life situations, the same behavior that such women would not reproach to DTM. But NAM (New Alternative Masculinities) are reacting to this. This article analyzes the communicative acts employed in all these situations, both those produced by women and DTM, as well as NAM’s communicative acts in response to those offenses. Data was collected using communicative daily life stories of give women and three men with diverse profiles and different levels of participation in women’s and men’s movements. Findings highlight, from the transformative dimension of the communicative methodology, that the use of language of desire in NAM’s reactions is effective not only to make justice with men who have never executed violence on women, but also to undermine the attractiveness of both DTM’s behavior and the comments of some women on such behavior. These findings complement previous research on preventive socialization of gender violence by broadening scientific knowledge on NAM’s communicative acts that prevent and eradicate gender-based violence. Further research ought to broaden the evidence of how some women who defend feminist values sometimes do not support and even tease or reprimand men who practice these values; moreover, an important line could analyze the way people talk about men with NAM attitudes to hold back reprimands in comparison to how people talk about men who follow a DTM model.

## Introduction

Xavi: I remembered an example of a communicative act shared in a meeting where we were talking about trends in New Alternative Masculinities (NAM). One researcher explained the situation of a woman who always was reprimanding her boyfriend (Abel) for not doing some domestic work. This boy had egalitarian values and was worried by that, thinking that he was worse than her ex-boyfriend (Mauro). Then, this researcher, who knew that her ex-boyfriend (who follows a DTM model) never did domestic work and had sexist attitudes toward her, said to this friend (Abel): “Come on! He has never cooked in his life!”

Xavi’s example is based on a communicative act performed by a researcher to his friend (Abel). His friend was worried about his girlfriend because she reprimanded him about domestic work. But the researcher knew that his girlfriend never reprimanded her ex-boyfriend (Mauro), who never did any domestic work and held attitudes belonging to the DTM model (Flecha et al., 2013), according to Xavi’s words. DTM stands for hegemonic men with non-egalitarian attitudes, the main perpetrators of gender-based violence. For this reason, the researcher stopped the effect of this reprimand to his friend (a man who never perpetrated gender violence) of seeing himself as worse than the DTM ex-boyfriend. This reprimand was stopped through the communicative act performed by the researcher who has a NAM attitude: “Come on, he has never cooked in his life!”, according to Xavi’s words. NAM are egalitarian men who portray strong, confident, and coherent attitudes in their relationships and are therefore a successful and attractive alternative against traditional models.

There are multiple studies related to the language used to talk about women that enable us to identify social inequalities and violence in gender relationships (Kollock et al., 1985; Jenkins and Aube, 2002; Holmes, 2006; Talbot, 2006; Boonzaier, 2008; Newman et al., 2008; Greenwood and Gautam, 2020). Language is a central tool in human life according to words Parodi’s (2009), and for this reason research on language and gender is studied by scholars from diverse disciplines, according to Talbot (2006). Academics from this research field have explored the use of language in diverse fields, for instance in representation in media, broadcasting, and other institutions including the family. However, literature related to the language used by women toward men who never perpetrated violence is scarce. Therefore, the analysis of women’s uses of language toward non-violent men in daily situations (such as Xavi’s example provided above) and the language used toward violent men have been detected as a research gap that needs to be filled. The present research is an exploration of this language used by some women toward the Oppressed Traditional Masculinities (OTM, hereinafter) model (Flecha et al., 2013). OTM include men who do not practice violence but who don’t awaken attraction, since they lack confidence. Particularly, we analyze which type of communicative acts (Santa Cruz and Redondo, 2010) are used to reprimand OTM or men who have never exercised gender violence in different daily situations: for instance, when they are not doing some domestic work, in daily work situations, or in leisure situations with friends; and which type of communicative acts are used by these same women addressed to Dominant Traditional Masculinities (DTM, hereinafter) in similar situations. Further, our research does not only focus on these language uses, but it also sheds light on the different types of responses provided by men. Particularly, we have identified which type of communicative acts performed by NAM (hereinafter) (Flecha et al., 2013) tend to eradicate any reprimand or joke about men who have never perpetrated violence.

In doing so, the structure of this article is composed of four sections. The first section presents a review of the main contributions from the specialized literature related to how reprimands, jokes and teasing are used by women and men in order to ridicule someone, for instance in what situations they are used, and which collective suffers this type of language use most. Second, the methodology used in this study, involving data collection, and analysis, is presented. Third, we introduce the main findings which have been classified in two main categories: exclusionary communicative acts that reprimand and ridicule OTM expressed by some women and DTM; and transformative communicative acts performed by NAM that react upon these reprimands. Finally, the main conclusions of the study are provided.

### Research on the Use of Reprimands and Teasing in Gender Relationships

Previous to this study, a literature review was conducted focused on identifying the use of reprimands and teasing used by women and men in daily situations. The evidence found has been grouped into two main bodies of literature. The first section introduces the analysis of reprimands, jokes and teasing used in daily situations. In the second one, those evidence that confirm how dominant men are who reprimand and tease men who are not violent and a trend of some women who are reprimanding and teasing men who are not violent are introduced. At the end of the review, we present the current challenge faced by research, that is, the need to explore how to overcome these types of reprimands through the positioning of NAM’s movement that counteracts these interactions, and how the present article is meant to represent a step forward toward this direction.

The first body of reviewed literature is focused on what we know about how sometimes reprimands, jokes, and teasing are used for ridiculing someone. In order to investigate more profoundly how they are used by people, some guiding questions have been defined to explore literature on heterosexual relationships. For instance, how are reprimands, teasing and jokes used for ridiculing? Who is using them? Addressed to whom? What are the consequences of that? Research in this field has already highlighted some meaningful information worth remarking upon here.

In the case of humor and jokes, milestone works have already pointed out how jokes exercise a double function: they are used to maintain healthy relationships and friendship, and to perpetuate power relationships (Hay, 2000; Holmes, 2006; McCann et al., 2010; Rees and Monrouxe, 2010; Abedinifard, 2016; Greenwood and Gautam, 2020). Our work is focused on the latter function. Evidence is provided by McCann et al. (2010) about how humor could be used to ridicule persons, such as jokes that are close to homophobia. They investigated about how these jokes could constrain the attitudes and behaviors of all men, how they are used for preserving what is considered to be a real heterosexual masculinity and what is not, McCann et al. (2010). This contribution is related to reflections of Jewkes and Morrell (2010); they point out that hegemonic masculinity not only maintains power over women, but also produces hierarchies among men, often marginalizing those that do not adhere to the dominant model.

Other scholars such as Holmes (2006) found that gender stereotypes are the focus of workplace humor. Holmes (2006) pointed out that sometimes men and women reinforce negative stereotypes in their informal conversations: for instance, men are treated as communicatively incompetent and women as sexual objects. In the same line, Rees and Monrouxe (2010) analyzed how laughter is a mechanism for constructing gender identities. Sometimes, this mechanism could damage people who are the object of it. In the authors’ words: “how laughter can make people feel bad (e.g., the butt of the tease)” (Rees and Monrouxe, 2010, 335).

Therefore, jokes, laughter, and humor could be used for hurting, and result in teasing mechanisms. In fact, Hay (2000) found that teasing was one of the languages used to maintain power over particular human groups. Specifically, Hay (2000) identified three functions played by humor in these settings: reinforcing solidarity, maintaining power-based relationships and psychological needs (to defend and to cope). Regarding the function of maintaining power-based relationships, Hay (2000) highlighted the aim of the teasing mechanism: “teases serve primarily to maintain the power of the teaser (Hay, 2000, 720)”. One of the questions emerged is “who is the teaser then?” And “who is the target of the teaser?” It is clear how humor plays a role in perpetuating power relations in a wide range of situations; however, to what extent is this also used to maintain power relations within gender relationships? While some research has highlighted the use of humor in reinforcing gender stereotypes, mainly targeting women, scarce efforts have been dedicated to analyzing in which ways some women (Gómez, 2015) are also using jokes and humor in order to discredit and exercise power over particular men. This leads us to the second body of literature.

Evidence found confirms how boys and men who follow the dominant men model are those who exercise this oppression (McCarry, 2010; Hamlall and Morrell, 2012). On the contrary, men who are far away from this dominant model are those who mainly suffer reprimands and teasing from some women and dominant men (Jenkins and Aube, 2002; Bergmann et al., 2014; Pinilla et al., 2014). Examples of these reprimands and teasing have been found in different daily situations in diverse spaces: school, social movements, and in sexual affective relationships.

In this sense, Hamlall and Morrell (2012) conducted a research with secondary students and identified how the dominant masculinity model influenced students’ interactions. Among other contributions, researchers highlighted that those boys who follow the dominant masculinity model were teasing other boys and the terms that are used:

Dominant boys at the school called certain boys “gays” and “sissies” as a way of marginalizing them, and at the same time elevating themselves and endorsing the misogynistic and homophobic elements of the school’s gender regime (Hamlall and Morrell, 2012, 492).

Due to this affirmation, dominant boys are learning to use teases for ridiculing others and maintaining their social status. The coercive dominant discourse (Puigvert and Flecha, 2018; Puigvert et al., 2019) influences these phenomena, emptying the attractiveness of the OTM men with such comments while reinforcing the link existing between dominant men and desire (Ruiz-Eugenio et al., 2020a; Racionero-Plaza et al., 2021). In other research, dominant young men are identified with those that are more prone to perpetrate violence against girls (McCarry, 2010). She found that young males with a violent attitude against others coincide with those young men who are ascribed to the dominant model and this was a significant factor of the perpetration of male abuse and violence against girls involved with them.

The following step was to explore what has been studied about how reprimands have been used by women and which type of masculine model was their target. Literature in this specific field is scarce and not many answers are found to this question in the literature reviewed. Nevertheless, some evidence has been collected that approaches the issue. Pinilla et al. (2014) conducted a qualitative research on men involved in the “Egalitarian Men Groups” in Spain and their relationship with feminists in order to analyze their role in the fight against gender-based violence and gender equality. Researchers found that some feminists who were in power positions established vertical relationships with these men with egalitarian values. Particularly, they showed how these men felt that they often had to give explanations to some feminists who represent the institutional movement and how some male colleagues assumed this submissive position (Pinilla et al., 2014), although they shared the same goals of gender equality and fight against gender violence. For this reason, some men interviewed did not understand why they were treated in this way. Our research will shed light on this phenomenon and how communicative acts which are apparently performed by some feminist standpoints can do otherwise and go against these ideals and even perpetuate gender violence.

Situations identified in research indicate how the use of reprimands and teasing are mainly addressed to those men who have not perpetrated gender violence. Evidence shows a trend of some women using reprimands and teasing against those men who are in favor of gender equality or are fighting against gender violence. However, there is no evidence in the literature whether the same women are using these reprimands toward those men who belong to the DTM. Thus, one of the challenges of this study is to deepen in the analysis of reprimands and teasing used by some women addressed to men who do not perpetrate gender violence, and to see if there is any evidence that the same women are also reprimanding and teasing those men that have perpetrated gender violence.

Concluding, situations as the ones described above indicate that some men are stigmatized for not following the DTM model although they are supporting women’s rights and gender equality, consistent with research results of Rudman et al. (2012). In this sense, Primer and Moss-Racusin (2009) said that it would be necessary to introduce changes in ways of talking about men that support gender equality: “If it was understood that men who are supportive of women’s efforts for equality are strong men—by definition [that] it actually takes more strength as a man—that changes the conversation” (Primer and Moss-Racusin, 2009, 15). This idea helps to search which type of leadership could contribute to transform practices (Santamaría and Jean-Marie, 2014). In this sense, NAM’s movement has achieved this perspective, by combining language of desire and of ethics in their communication. In doing so, they are attracting young men to their movement because they show that it is possible to be committed against gender violence (Joanpere and Morlà, 2019) while not allowing for being undervalued (Serradell et al., 2014). Following the research by Puigvert et al. (2019), NAM are not submissive to that coercive dominant discourse that separates goodness from success and attraction (Ruiz-Eugenio et al., 2020b). Due to the positioning of the NAM’s movement, it has been considered suitable to collect examples of reactions of NAM to counteract reprimands and teasing addressed to men who do not perpetrate gender violence. The last aim is to analyze what types of communicative acts are used to counteract reprimands and overcome inequality in order to cover an aspect that is not present in the literature, departing from the previous theoretical contributions.

## Materials and Methods

The study has been carried out using the communicative methodology (Flecha and Soler, 2014; Gómez, 2019). One of the main characteristics of this methodology is the creation of dialogic knowledge. This is possible because researchers and participants establish an egalitarian dialog where the former bring into the conversation the accumulated scientific knowledge in order to contrast it with the latter’s lifeworld (Ruiz-Eugenio et al., 2020a). Besides, the present study has also been framed under the communicative acts theory (Santa Cruz and Redondo, 2010) focusing on the conflictive interactions developed in everyday life situations dealing with domestic chores, daily work situations, and leisure situations with friends and in partnership. Communicative acts include the analysis of verbal and non-verbal language, the influence of the social context where interactions occur, and the intentions and responsibility of the consequences of the whole communicative act that is performed (Rodríguez-Navarro et al., 2014). As Itakura (2014) concluded, it is necessary to include non-verbal elements and context for analyzing conversations in depth.

### Data Collection

This study focused on communicative acts that reprimand OTM undertaken by some women or DTM, and communicative acts performed by NAM that counteract reprimands. To start the interview, researchers explained both frameworks, communicative acts (Santa Cruz and Redondo, 2010) and the different types of masculinity OTM, DTM, and NAM (Serradell et al., 2014), in plain language to the participants. This presentation was the starting point of a shared reflection in which participants were asked if they knew any situations like the ones described in the results’ section and whether they could provide personal examples or cases of their friends or colleagues. On the one hand, they were asked to explain communicative acts of women or DTM men, specific things they did or said toward a man that corresponds to the OTM as a reprimand, with bad manners, mocking, comments with second intentions that show complaint or disdain, in reply to some daily life attitudes of that man, such as at work, with friends, or at home. On the other hand, they were asked to explain communicative acts from NAM that respond to the former, where a language of desire is present, in the search of justice toward such non-violent men who are targets of the reprimands, and also to empty of attractiveness such comments. The data presented in this paper was collected from five communicative daily life stories with women, and three communicative daily life stories with men. This technique allows to go deeper into the participants’ past and present events and to discuss the analysis of the interpretation of these events. Each daily life story lasted around 45 min. All participants were informed about the aim of the research before signing the informed consent. Pseudonyms (see Table 1) are used in order to preserve their anonymity and privacy. The consent forms comply with the international standards established by the European Framework Programme (European Commission, 2019).

**TABLE 1:** Participants’ profile.

**N**	**Pseudonym**	**Age**	**Profile**
1	Ana	50–55 years old	Educational advisor
2	Alejandra	60–65 years old	Educational advisor
3	Begoña	50–55 years old	Teacher of secondary school
4	Lucía	40–45 years old	University professor
5	Laia	35–40 years old	University professor
6	Marc	35–40 years old	University professor
7	Luis	45–50 years old	Factory worker

### Criteria and Selection of Participants

We have selected 8 participants (5 women’s and 3 men’s), people who would be able and willing to share meaningful communicative acts on the topic studied. The basic criteria for the selection were aimed at achieving a certain level of diversity, as shown in different Socioeconomic status (SES) levels, age, gender, and family status (single, married, with a partner, etc.). A second basic criterion that came up in the literature was to interview people with different levels of involvement in women’s and men’s movement. According to evidence analyzed, examples of reprimands and teasing are mostly known by people involved in feminist movements or men groups (Bergmann et al., 2014; Pinilla et al., 2014). The participant’s profile is the following:

Ana, Alejandra, and Begoña have a historical perspective of the changes that have occurred in gender relationships since the end of the seventies. Ana is more involved in educational practices that are working to overcome gender violence. Alejandra and Begoña share the common situation of having women friends involved in feminist movements but they are not directly involved. Lucia and Laia are both university professors and they have participated in different social movements; specifically, they have been activists in diverse feminist movements. Marc and Xavi are university professors, and they are directly involved in NAM’s movement. Last but not least, Luis is working in a factory and is not involved in any movement.

### Data Analysis

First, concrete communicative acts that best represent the daily life stories were selected, in order to analyze communicative acts performed by women and men toward OTM that exemplified reprimands and teasing of how they do something (at home, work, etc.). Second, communicative acts expressed by NAM that counteract teasing and reprimand were analyzed. Thus, two main dimensions were defined: exclusionary communicative acts that reprimand, and transformative communicative acts that counteract reprimands. In order to analyze these examples, the conversations were analyzed according to the communicative acts’ theory (Santa Cruz and Redondo, 2010). For this purpose, the focus of the analysis followed two dimensions of verbal and non-verbal language; one, that exemplifies the exclusionary dimension (reprimands and teasing linked to violence, and silence/non-reaction in front of these situations) and, two, the transformative dimension (counteracting reprimands and teasing/ethic and desire linked to non-violence) (Ruiz-Eugenio et al., 2020a).

## Results

Based on the communicative data analysis, findings are grouped in two main sections. In the first one, those communicative acts that have been categorized as exclusionary as examples of reprimands and teasing addressed to men who never perpetrated gender violence; in the second, communicative acts performed by men who counteract reprimands addressed to men who have not perpetrated gender violence. The latter are considered to be transformative communicative acts.

### Exclusionary Communicative Acts: Reprimands and Teasing Addressed to Men Who Never Perpetrated Gender Violence

Six communicative acts have been selected from the fieldwork: among them, four communicative acts are examples of reprimands and teasing toward non-violent men performed by women, and two communicative acts performed by men that follow DTM model who are teasing other men who do domestic work and who do not perpetrate gender violence.

The data collected through the communicative daily life stories provide evidence about how some women usually reprimand OTM or men who never have exercised gender violence for issues related to domestic work. For instance, when talking about these issues, Lucia began to remember examples of her daily life that illustrate these situations. Specifically, Lucia provided examples of reprimands performed by women addressed to their partners (and these men were not violent) regarding domestic work issues. From these examples, we have selected two concrete communicative acts. Lucia for instance explained a reprimand of one friend to her partner because he was not folding the clothes in the way she wants it, reacting to this with a scream:

You are putting the thicker clothes inside the drier and then they are drying slowly, look at this! Or when he is cooking she said; You leave the kitchen a mess when you cook!

As Lucia remembered this example, she added that this same friend did not make any reprimands to her ex-boyfriend who never did any domestic work and who had a sexist attitude with her. This comment of Lucia indicates a first discovery that was not found in the literature: the same woman, who reprimands her partner for doing domestic work in his style, did not reprimand her ex-boyfriend that did not do any domestic work and treated her with a sexist attitude.

During the conversation with Lucia, she explained that these daily situations are common with all domestic tasks and also with childcare in some circles of friends. An example of this is that of a friend of hers who considers that her partner does not know how to properly dress their children:

I do not understand how you do not differentiate the right and wrong sides of the dress! How can you put it inside out?

In this sense, these types of interactions help to reflect about what type of language hinders the advance of egalitarian relationships between men and women. Hearn (2006) highlights the importance for men to be more active in domestic work and childcare for achieving more equal societies in Europe. But if some women reprimand their partners for their domestic work, it is not possible to advance in this direction, as they are emptying their relationship of attractiveness and breaking the sense of it.

The next two communicative acts selected are examples of reprimands made by women from different spaces. The first one is an example of how a girl reprimands her boyfriend in front of her group of friends, ridiculing him publicly. The second example shows how reprimands can occur in daily work situations.

Laia, a woman of 38 years, when asked if she knew examples of communicative acts of reprimands performed by women addressed to their (non-violent) partners, immediately recalled a situation of a couple (Noelia and Juan) who went to the beach at night with a group of friends, and how she always used to reprimand him even in front of their friends:

Laia: I remember a couple where Noelia treated her boyfriend Juan badly. He was very nice with her. One day a group of friends went to the beach at night (…) and she said that she was cold, then her partner (Juan) without saying anything went to the car (it was very far from us) to find a jacket for her. When Juan came back, he put the jacket on her by surprise and Noelia said; “Fuck! Why are you giving me this jacket? I don’t like it, why do you go to get this jacket, I didn’t tell you to do it! You are an idiot!” – I was impacted, and Noelia said many times; “We make love only for his birthday and at the end of the year, and not more” – I remember that I talked to Juan and I told him that it would be better for him not to continue with Noelia, and he told me “It is ok for me, I have no personality”.… Time passed and Noelia left him for a DTM boy, and she never treated this new boy in the same way that she did with Juan.

This communicative act proves that Noelia does not reprimand all men, but only a particular type: the ones who she feels are inferior to her. According to Laia’s words, Juan represents the OTM model, and Noelia often reprimands him. However, Laia identified that Noelia’s current boyfriend belonged to the DTM model, and Noelia never reprimands him as she did with Juan. In this example of communicative acts, the language and the tone used by Noelia are exclusionary because she uses an abrupt language (“Fuck, why are you giving me this jacket?”), using insults (“you’re an idiot”), and emptying his social image from any sexual attraction in front all of the rest of people who were living this situation (“We make love only for his birthday…”). This kind of interaction is evidence of how this girl has assumed a DTM model and she reproduces it with Juan. This evidence is linked with the result of Jenkins and Aube (2002) and Gómez (2015), who show how some women were reproducing attitudes and habits of the dominant masculinity model. The communicative act of Juan is exclusionary because he is not reacting in front of his girlfriend’s reprimands, he stays silent, and when Laia asked him why he is still with Noelia, who is not treating him well, his answer is to assume his submissive role with an insecure tone (“It is ok for me, I have no personality.”). This man’s reaction is consistent with the research conducted on how some men perpetuated the unfair and violent treatment because they are tolerating it (Talbot and Quayle, 2010; Messerschmidt, 2012; Flecha et al., 2013).

The fourth act of communication selected exemplifies an interaction which occurred in the workplace. Begoña, a 55-year-old woman, remembered during her life story various situations within teams of teachers in schools. Begoña is an educational advisor, and therefore knows firsthand the interactions that take place in teachers’ meetings. Begoña told us how some female colleagues of a secondary school changed their behavior toward a new coming physical education teacher (Mario). In the words of Begoña, these women initially thought he was the typical cocky guy, and considered him very attractive, but Begoña explains that as Mario is showing his sensitivity and romanticism toward his current partner (Maria), the same colleague who had considered him attractive, began to tease him.

Begoña: I’m thinking of a secondary teacher (Mario)…. He had a strong body… and all the school teachers where I’m the advisor considered him physically attractive during the first days, they said “He is hot! mmm”…. But when Mario shared that he was in love with his partner (Maria) and he proposed marriage to his girlfriend giving her a nice ring belonging to his family, interactions of these women teachers began to change. Mario usually explained how he cares for his partner and his friends, sharing the attention he has for them and also gifts that he received from his partner and friends. Then, his female colleagues began to criticize him by saying “Look at him! He is like a woman,” and “he is a child and not a man, too soft, too healthy” (because he eats apples instead of drinking wine, etc.) … I think that these women envied him. Yes, this is an example of how this boy was considered attractive and how these women emptied his attractiveness doing reprimands and ridiculing him because he shared beautiful feelings and was an egalitarian man.

This communicative act is an example of how some women use a type of language of desire (“He is hot! Mm”) in relation to a man that is physically attractive to them but whose communicative acts change when these women perceive that he is nice (Puigvert et al., 2019) and passionate with his wife and friends. When this occurs, these women begin to use communicative acts with no desire regarding that man and make jokes, trying to ridicule him (“Look at him! He is like a woman,” “he is a child and not a man, too soft, too healthy”). Begoña explained that this man was not worried about these comments; he continued with his healthy life and with his passionate relationship.

To conclude this section, we have selected two communicative acts performed by men who follow a DTM model addressed to men with egalitarian values and who have not perpetrated violence according to the participants interviewed. The first is provided by Alejandra, a 61-year-old woman, who told us she had identified such interactions especially in circles of friends. Specifically, she remembered a couple where Blanca and Antonio shared housework, but that shared responsibility was not well regarded by some friends of the couple and told us a situation where Antonio was teased by their friends for it.

Alejandra: They shared domestic work (Blanca and Antonio). I have a friendship with this couple, and I remember they have the agreement to share equally these tasks. Then I remember that he ironed the clothes…and he is a handsome man, and other men of the friends’ group, I think that they were DTM, were making fun of Antonio, for instance when they were drinking together, they said: “Where is Antonio? He’s probably ironing (hu, hu, hu)” and they all laughed at him.
Researcher: and the women. What did they say?
Alejandra: the women did not say anything when they were with these men in the group to defend this man, although they liked his attitude.

This communicative act illustrates how men tease Antonio because he was sharing domestic work with his wife. The exclusionary dimension of teasing is exemplified when they laugh about him (“Where is Antonio? He’s probably ironing (hu, hu, hu)”). This communicative act is an example of how laughter can be used as a tease, a mechanism that can hurt people who are the object of laughter, as Rees and Monrouxe already pointed out (2010). Additionally, the passive attitude of women in this situation, of not saying anything when the DTM men laughed and ridiculed the man who irons, even though they apparently liked the attitude of Antonio, is identified as an exclusionary dimension of this communicative act. Silence is also a mechanism of consenting to teasing and violence. Therefore, in this communicative act we can see how the silence of these women made them accomplices of the men who ridiculed Antonio, without reacting to this situation and allowing it to continue happening.

The last selected communicative act is provided by Laia again. When asked whether she knew any examples of communicative acts by some men who corresponded to the DTM model toward a man who was not, Laia confirmed and focused on describing the reaction of a boy who, according to her, corresponds to DTM (Julio) in front of a man (Manu) with egalitarian values toward women. In particular, Laia pays more attention to Manu than to Julio:

Laia: When I was younger, I usually went with a group of friends to the beach. One of my friends (Manu) was very nice and I liked to talk to him because I enjoyed it very much. One day my group went to the beach and I was talking with Manu and not with the DTM boy of our group (Julio) because I found him boring. Then, we were sitting at the beach and when some of us got up from the sand he [Julio] said “Look! I have left a hole in the sand with my ass”… Then all of us began to get up and see the holes that we had left… but my friend [Manu] would not do it …. Then Julio said “Look! This idiot doesn’t get up because he is fat… hu, hu, hu… and his ass…. sure he left a big hole… hu, hu, hu”. He was teasing him so much. And nobody was saying anything to him… finally some people told him to leave him alone…. And that it’d be better to get back to our motorbikes. Manu did not get up, he was alone there, because all of us went back and nobody stayed with him, we left him alone…. Now that I remember this, I think that it was a tough situation for him, and we should have been with him and respond to Julio more clearly.

This communicative act is a clear example of how DTM men need to ridicule men who do not represent hegemonic masculinity in order to maintain their power status inside the group, as Hay (2000) mentioned in her research. In fact, Julio (DTM) felt jealous, according to Laia’s words, because girls were not paying attention to him, and she preferred to talk with Manu. Julio needed to attack Manu with personal details (in this case physical traits) to reinforce his social status (“Look! This idiot would not get up because he is fat… hu, hu, hu… and his ass…. Sure he left a big hole… hu, hu, hu”). Dominant boys who are teasers usually use language to humiliate (Hamlall and Morrell, 2012) and this is an example of that.

### Transformative Communicative Acts: Courage and Attractiveness in NAM’s Reaction

This section exemplifies three communicative acts performed by men who follow NAM behavior, which undermines the attractiveness of DTM and women who try to ridicule OTM or men who never perpetrated gender violence. As we will show in our data, these communicative acts bring in a transformative dimension because they use a language of desire to counteract reprimands and teasing. We have selected communicative acts that occurred in different social contexts, evidencing that these interactions are present in diverse spaces. These three communicative acts (one personal and two from other two colleagues) are provided by Marc, an active member of the NAM movement.

The first communicative act selected occurred during a conference organized by the “Men in Dialogue” association in Barcelona in 2013. After finishing the conference an activist (Jose) who explained successful strategies to prevent gender violence, a woman from the public addressed Jose to tell him how men’s movements have to organize their fight. Marc vividly revealed this situation during his communicative daily life story:

Marc: In the Q&A, a woman from the floor started to tell us in a very authoritarian tone “Men have to do this and that.” Jose replied: “Men will decide what we should do.” He said it with self-confidence, and he emptied the attractiveness of the woman. We responded instead of shutting up.

The literature review evidenced how some men are afraid to answer this type of reactions, especially if they come from well-established feminist women (Pinilla et al., 2014). Our analysis shows how NAM are not afraid to be assertive and respond to the communicative acts that try to dominate them (Serradell et al., 2014). They work together with feminists who treat them in an egalitarian way, not from a power-based position. In the same way that feminist movements considered as an imposition that men would decide about aspects concerned to women’s life, these men also refuse any type of imposition that some women might want to impose on men’s issues. From our data, it can be inferred that an effective way to respond to the communicative acts that try to dominate men is by having a secure attitude as Jose said: (“Men will decide what we should do”).

The second communicative act selected is a personal situation experienced first-hand by Marc. When Marc worked at a technology company, he remembered one colleague, in particular, representing the DTM model (Roberto). According to Marc, Roberto used to ridicule those men who did not follow his model (Racionero-Plaza et al., 2021) as well as making very derogatory comments toward women. As the company had partners in different European countries, the example provided by Marc refers to a night out of the team after a meeting in Belgium. Roberto tried to persuade all the team to enter a brothel appealing to their masculinity, according to Marc, pressing and teasing who would not want to go there.

Marc: I lived this situation when I was with my colleagues and some clients on a men’s night out. Roberto (DTM) wanted to joke and said we had to go to this brothel, pretending to be funny and boasting of his masculinity.
Researcher: Really?
Marc: Yes. I said that I didn’t want to go inside, because I don’t like it and also because of my ethical principles. There, Roberto tried to tease me, but he didn’t achieve to make me feel bad and he didn’t change my decision. Instead, the boss (Albert) of our team supported my decision and said that he would do the same. Albert was a leader, apart from being the boss, and he decided to stop the social pressure imposed by Roberto and he didn’t go inside the brothel.
Researcher: But he did this because you were the most courageous, maybe he thought “hopefully someone says not to go inside…”
Marc: Yes, that’s it. Roberto was mocking, and the boss and I felt fine with our positioning. We felt complicity and others left the brothel quickly; Roberto remained in ridicule.

According to Marc’s narrative, Roberto tried to impose on the group to go to a brothel, deciding that it is the nature of heterosexual masculinity, using humor and jokes to convince them. This interaction is an example of how dominant men decide what a “real man” is (McCann et al., 2010). However, Marc decided to say “No” to going to this brothel because he did not like it and for his ethical principles. This is an example of a communicative act that includes desire and the language of ethics. He transmitted self-confidence and the boss reacted and supported his decision. This is evidence of the link between NAM and leadership toward social change already stated by Redondo (2016). Marc said that the boss and he had a good night talking in another bar and developed a good mutual understanding; from this moment their friendship grew. This evidences how male friendships who share a clear statement against gender violence (in this case a clear position for not wanting to go to a brothel) generated a better and passionate friendship (Gomez, 2014). The courageous attitude of Marc was crucial for not being an accomplice and to help his boss to be courageous too. This situation put DTM in ridicule, emptying his attractiveness. Robert lost the power in this situation in front of the group, and the possibility to decide what a “real man” is. In fact, we have real heroes in diverse social contexts, the only thing that we should do is to explain more often this type of communicative acts and counteract the dominant discourse, as Primer and Moss-Racusin (2009).

The last communicative act selected refers to Lucas, Marc’s friend, who works as a teacher in a high school. Marc told us about how a colleague of Lucas tried to ridicule him when he saw that Lucas was receiving romantic messages from his wife. This situation occurred in the teacher’s room of a secondary school:

Marc: One male teacher saw chat messages of Lucas’ wife “How are you, my love?” This teacher tried to ridicule Lucas and said: “You are a soft man, do you allow that your wife tells you this “how are you, my love?” Then Lucas told him “Hey man! Don’t blame me if your wife doesn’t tell you these things but tells you to go to the couch!”

This last example evidences how Marc was sharing the importance of Lucas using the language of desire and not consenting to be ridiculed. The interaction of the colleague tried to ridicule Lucas. But Lucas, instead of shutting up, responded to this situation with the language of desire (“Hey man! Don’t blame me if your wife doesn’t tell you these things but tells you to go to the couch!”). In this way, Lucas achieved to empty the attractiveness of that man who is teasing him.

## Discussion

Our research has shed light on how DTM and some women reprimand or ridicule OTM or men who never have perpetrated gender violence. We have also shown how OTM oftentimes do not effectively respond to these exclusionary communicative acts. Instead, some OTM tolerate it, thus perpetuating unequal relationships. Our data also indicates how some women are accomplices of this type of communicative acts. Evidence shows that they only reprimand OTM but not DTM, thus strengthening the coercive dominant discourse that moves away attraction from kindness (Puigvert et al., 2019). The communicative acts analyzed here illustrate that men who follow a NAM model counteract these communicative acts and overcome them (Rodríguez-Navarro et al., 2014). They use a language of desire for undermining the attractiveness of DTM and women who reproduce that behavior; in fact, they are achieving and changing the dominant discourse, releasing their circles from the coercion by practicing communicative acts that promote a freer socialization based on egalitarian values combined with a language of desire. This language used represents a real alternative to combat gender-based violence and to achieve better relationships where nobody is being subordinated to anyone, and where more possibilities to achieve satisfactory relationships are given; where communicative acts are free of unjust reprimands.

Our findings lead to the identification of several implications for further research. First, the need to go deeper into the evidence of how some women who defend feminist values, in some cases do not support men who practice these values, in some cases they are even reprimanding or teasing them in public. However, in the cases analyzed this same behavior is not reproduced in interaction with those men who represent the dominant DTM model. This fact evidences incongruences between discourse and practice of these women. Hence, this result could be included in feminist debates that could help to advance in the aim of constructing egalitarian relationships between women and men and to review the coherence between discourse and practice in gender relationships.

Second, this article has been a first step to reveal how communicative acts performed by NAM counteract reprimands done by some women and men who follow the DTM model. They turn the situation around, and these women and men are dismantled. Another crucial factor obtained is the reaction of those people who experience these communicative acts of teasing. In this sense, future research could analyze how people talk about men with a NAM attitude counteracting reprimands and how people talk about men who follow the DTM model. Thus, the analysis of the impact of NAM’s communicative acts in the perception of those people who are present during the reaction of NAMs could be collected to evidence who is more valued and who is ridiculed. According to our initial data, DTM are considered ridiculous after communicative acts performed by NAM (Rodríguez-Navarro et al., 2014); thus, violent attitudes are undermined. Therefore, further research is needed in this area because this result is crucial for preventing gender-violence relationships.

Limitations of this study mainly refer to the fact that communicative acts are basically examples of past events, and some of them from third persons. This implied a certain level of difficulty because the research participants had to remember concrete situations and some specific details might not be exact. To reduce this limitation the researchers consistently posed follow-up questions that helped the participant to remember relevant details related to verbal, non-verbal and interactions from those persons who participated in the communicative act.

## Data Availability Statement

The raw data supporting the conclusions of this article will be made available by the authors, without undue reservation.

## Ethics Statement

The studies involving human participants were reviewed and approved by CREA, Community of Research on Excellence for All. University of Barcelona. The patients/participants provided their written informed consent to participate in this study.

## Author Contributions

RV-C approached the article’s conceptualization. AM-P contributed to some insights on the literature review. RV-C and GL-T prepared and revised the final version of the manuscript. BM reviewed the last version of the manuscript. All authors contributed to the article and approved the submitted version.

## Conflict of Interest

The authors declare that the research was conducted in the absence of any commercial or financial relationships that could be construed as a potential conflict of interest.
